# Biogenesis of RNA Polymerases in Yeast

**DOI:** 10.3389/fmolb.2021.669300

**Published:** 2021-04-28

**Authors:** Ana I. Garrido-Godino, Francisco Gutiérrez-Santiago, Francisco Navarro

**Affiliations:** ^1^Departamento de Biología Experimental-Genética, Universidad de Jaén, Jaén, Spain; ^2^Centro de Estudios Avanzados en Aceite de Oliva y Olivar, Universidad de Jaén, Jaén, Spain

**Keywords:** RNA polymerases, biogenesis, assembly, transcription, yeast

## Abstract

Eukaryotic RNA polymerases (RNA pols) transcriptional processes have been extensively investigated, and the structural analysis of eukaryotic RNA pols has been explored. However, the global assembly and biogenesis of these heteromultimeric complexes have been narrowly studied. Despite nuclear transcription being carried out by three RNA polymerases in eukaryotes (five in plants) with specificity in the synthesis of different RNA types, the biogenesis process has been proposed to be similar, at least for RNA pol II, to that of bacteria, which contains only one RNA pol. The formation of three different interacting subassembly complexes to conform the complete enzyme in the cytoplasm, prior to its nuclear import, has been assumed. In *Saccharomyces cerevisiae*, recent studies have examined in depth the biogenesis of RNA polymerases by characterizing some elements involved in the assembly of these multisubunit complexes, some of which are conserved in humans. This study reviews the latest studies governing the mechanisms and proteins described as being involved in the biogenesis of RNA polymerases in yeast.

## Introduction

Transcription is the most studied step of the gene expression catalyzed by RNA polymerases (RNA pols). Eukaryotes contain at least three RNA pols (RNA pols I, II, III), while archaea and bacteria consist of a single enzyme (Werner and Grohmann, 2011). In addition, two additional RNA pols have been described to be present in plants (RNA pols IV and V) (Wierzbicki et al., 2008; Haag and Pikaard, 2011; Haag et al., 2014). Although single-subunit RNA pols exist (as in bacteriophage T7) (Kwapisz et al., 2008), the bacterial, archaeal, and eukaryotic RNA pols are heteromultimeric complexes (Werner and Grohmann, 2011; Cramer, 2019). In eukaryotes, RNA pol I comprises 14 subunits and synthesizes a precursor of the three largest rRNAs (Werner et al., 2009; Lane et al., 2011; Werner and Grohmann, 2011; Moreno-Morcillo et al., 2014). RNA pol II contains 12 subunits and is responsible for the transcription of mRNAs and some non-coding RNAs (Armache et al., 2003, 2005; Werner and Grohmann, 2011). RNA pol III is composed of 17 subunits and catalyzes the synthesis of tRNAs and 5S rRNA, as well as other non-coding RNAs (Werner et al., 2009; Fernández-Tornero et al., 2011; Lane et al., 2011; Werner and Grohmann, 2011; Dieci et al., 2012; Khatter et al., 2017).

Despite the fact that the transcription process and regulation have been extensively studied along with the structure of RNA pols (Ishihama, 1981; Briand et al., 2001; Cramer, 2002, 2019; Armache et al., 2003, 2005; Werner, 2007; Werner et al., 2009; Fernández-Tornero et al., 2011, 2013; Lane et al., 2011; Werner and Grohmann, 2011; Moreno-Morcillo et al., 2014), very little is known about the biogenesis of multisubunit RNA pols and how these processes occur in yeast. Several studies have identified many factors involved in the assembly and/or nuclear transport of RNA polymerases in both yeast and human cells, with most of them operating for the biogenesis of RNA pol II. In light of this, this review focuses on pre-existing knowledge of the assembly of RNA polymerases in yeast.

## General Overview of the Assembly Processes of RNA Polymerases

A model for bacterial RNA pol (ααββ’ω subunits) assembly has been proposed based on *in vitro* experiments. Assembly would start with the formation of the αα dimer, which would interact with the β subunit. Later, the ααβ module would associate with the β’ subunit, which probably forms a complex with the ω subunit (Ishihama, 1981). Interestingly, the ω subunit, which is not essential, seems to stabilize the β’ subunit (Minakhin et al., 2001).

In yeast, a model for the biogenesis of RNA pol II based on bacterial RNA pol formation (Ishihama, 1981) has been suggested (Wild and Cramer, 2012). A similar model has been proposed in human cells, which suggests the conservation of these processes. Yeast RNA pol II possesses the bacterial homolog ααββ’ω core composed of subunits Rpb1, Rpb2, Rpb3, Rpb11, and Rpb6 (Zhang et al., 1999; Werner and Grohmann, 2011; Wild and Cramer, 2012; Cramer, 2019). These subunits are conserved in yeast RNA pol I and III (Werner and Grohmann, 2011; Cramer, 2019). Accordingly, the Rpb3 subassembly complex (corresponding to αα dimer: Rpb3, Rpb10, Rpb11, and Rpb12) would form and interact with the Rpb2 subassembly complex (similar β subunit: Rpb2 and Rpb9) prior to the association with the Rpb1 subassembly module (β’ω subunits: composed of Rpb1, Rpb5, Rpb6, and Rpb8). Rpb6 (ω, subunit) would act by stabilizing the largest subunit of RNA pol II for the assembly of RNA pol II (Nouraini et al., 1996; Minakhin et al., 2001; Garrido-Godino et al., 2013) in line with the role proposed for the ω subunit (Minakhin et al., 2001). Finally, the stalk subcomplex (Rpb4/7) would associate with the preassembled core enzyme (Wild and Cramer, 2012 and our unpublished data), although it can dissociate (Armache et al., 2005).

While the proposed model accounts for RNA pol II, the question of what occurs for RNA pol I and III arises. By taking into account the conservation of the different subunits ααββ’ω in RNA pol I and III, similar pathways may act for the assembly of all eukaryotic RNA pols, and this process is likely coordinated by the existence of five RNA pols common subunits: Rpb5, Rpb6, Rpb8, Rpb10, and Rpb12 (Wild and Cramer, 2012). It is worth noting that the yeast homologous ω subunit Rpb6 has also been described to stabilize RNA pol I in addition to RNA pol II (Nouraini et al., 1996; Minakhin et al., 2001; Garrido-Godino et al., 2013). Furthermore, the availability of the shared subunit Rpb12 must be a limiting step in the assembly of the three RNA pols in yeast (Rubbi et al., 1999; Wild and Cramer, 2012). In addition, homologous Rpb4/7 complexes exist for RNA pol I and III (Rpa14/Rpa43 and Rpc17/Rpc25, respectively) (Werner and Grohmann, 2011). Nevertheless, the mechanisms governing the assembly of RNA pol I and III are described in another article published in the same issue by Boguta and Turowski (in press).

In line with previously proposed mechanisms for yeast, quantitative proteomic analyses in human cells have demonstrated the existence of a cytoplasmic RNA pol II subcomplex formed by subunits RPB2, RPB3, RPB10, RPB11, and RPB12 (Boulon et al., 2010), which suggests that the interaction of the RPB2 and RPB3 subassembly complexes may occur prior to the association with the RPB1 subassembly complex.

Another important question relates to where the assembly of the RNA polymerases occurs and how they enter the nucleus. RNA pol II assembly has been proposed to occur in the cytoplasm before its nuclear import in yeast (Boulon et al., 2010; Corden, 2011; Wild and Cramer, 2012; Mirón-García et al., 2013; Gómez-Navarro and Estruch, 2015), as it similarly occurs in human cells (Boulon et al., 2010; Corden, 2011; Wild and Cramer, 2012; Mirón-García et al., 2013; Gómez-Navarro and Estruch, 2015), and as it is suggestes by both the cytoplasmic accumulation of RNA pol II subunits after blocking biogenesis and by the identification of RNA pol II transport factors (i.e., Iwr1 and Rtp1) suggest (Czeko et al., 2011; Gómez-Navarro et al., 2013). However, additional Iwr1-independent mechanisms have been proposed to allow some RNA pol II subunits to passively diffuse into the nucleus in both yeast and human (Boulon et al., 2010; Gómez-Navarro and Estruch, 2015). Notably, cytoplasmic biogenesis has also been proposed for yeast RNA pol I and III based on the cytoplasmic accumulation of the largest subunits of their RNA pols under impairing assembly (Mirón-García et al., 2013). Although the mechanisms governing the assembly of RNA pol I and III are not as clear, mass spectrometry approaches and dissociation studies of the elongation complexes in yeast have identified the disassembly of RNA pol I and III and probably assembly subcomplexes (Schneider and Nomura, 2004; Lane et al., 2011). In yeast, the RNA pol III core may be assembled in the cytoplasm (Hardeland and Hurt, 2006; Mirón-García et al., 2013), whereas additional subcomplexes or free subunits must bind the core in the nucleus (Hardeland and Hurt, 2006). In fact, there are reports informing that the cytoplasmic accumulation of the second largest subunit Rpc128 also leads to the accumulation of other subunits, such as Rpc160, Rpc53, and Rpc11, whereas others remain nuclear (Hardeland and Hurt, 2006). Interestingly, dissociation analyses by mass spectrometry have evidenced that several subcomplexes appear after disturbing the RNA pol III structure *in vitro*, including stable trimer Rpc31/82/34 and the two heterodimers Rpc82/31 and Rpc17/25, as well as some free subunits like Rpb10, Rpc11, Rpc82, and Rpc34 (Lane et al., 2011). Notably, human RNA pol I assembly has been proposed to be sequential, but highly inefficient, even *in vivo*, with individual subunits entering the nucleolus rather than the preassembled holoenzyme (Dundr et al., 2002).

## Assembly Factors Are Required for the Assembly Processes of RNA Polymerases

**FIGURE 1:**
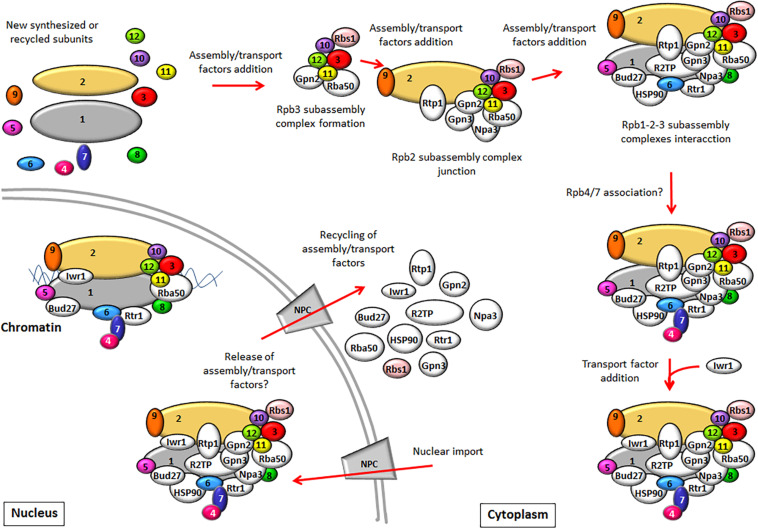
The biogenesis model of RNA pol II (RNA pol I and III assembly has been proposed to be similar). RNA pol II is composed of several subassembly complexes that sequentially interact to form the whole enzyme. The Rpb3 subassembly complex is formed prior to its interaction with the Rpb2 subassembly complex. Later, the Rpb1 subassembly complex junction leads to the core formation of RNA pol II. The association of the Rpb4/7 dimer with the rest of the complex probably occurs at a later step in the cytoplasm, although nuclear association must not be ruled out. RNA pol assembly requires the participation of assembly factors. After the core formation of RNA pol II, some assembly factors could be released, while others could participate in RNA pol II import, such as Npa3 or Rtr1. Import factor Iwr1 binds the active center of the full enzyme prior to its nuclear import. In the nucleus, assembly and import factors release RNA pol II and could be recycled to the cytoplasm, while others like Rtr1, Bud27, and Iwr1 could continue to be associated with RNA pol to mediate a role in transcription. The assembly factors described in yeast (some with human counterparts that play similar roles) are shown in white. Rbs1 is depicted in pink because it has been shown to participate only in the assembly of RNA pol III.

In yeast, the assembly of RNA pols and their transport to the nucleus require the action of assembly and/or transport factors, most of which are conserved in human cells. The pre-existing knowledge about their role in the assembly of yeast RNA pols and their comparison with that in humans are summarized in Table 1. How these factors act to mediate the sequential assembly of RNA pols and the nuclear transport is shown in Figure 1.

**TABLE 1:** Assembly factors.

**Assembly factor**		**Eukaryotic RNA pol**	**References**
***S. cerevisiae***	**Human**		
R2TP complex	R2TP/prefoldin-like complex	RNA pol II. Also suggested for RNA pol I and III	Boulon et al., 2010
Hsp90	HSP90	RNA pol II. Also suggested for RNA pol I and III	Boulon et al., 2010
Npa3	GPN1/RPAP4/XAB1/MBD*in*	RNA pol II	Forget et al., 2010; Staresincic et al., 2011; Niesser et al., 2015
Gpn2	GPN2	RNA pol II and III	Staresincic et al., 2011; Minaker et al., 2013; Zeng et al., 2018
Gpn3	GPN3/Parcs	RNA pol II and III	Calera et al., 2011; Minaker et al., 2013; Liu et al., 2020
Rba50	RPAP1	RNA pol II	Jeronimo et al., 2004; Zeng et al., 2018; Liu et al., 2020
Rtr1	RPAP2	RNA pol II	Forget et al., 2013; Gómez-Navarro and Estruch, 2015
Bud27	URI	RNA pol I, II, III	Mirón-García et al., 2013; Vernekar and Bhargava, 2015
Rtp1		RNA pol II	Gómez-Navarro et al., 2013
Rbs1		RNA pol III	Cieśla et al., 2015
Iwr1		RNA pol II	Czeko et al., 2011

### R2TP/Prefoldin-like

R2TP was initially identified in yeast as an Hsp90- associated multiprotein complex (R2TP-Hsp90 complex) (Zhao et al., 2005). This complex is well conserved from yeast to humans (Zhao et al., 2005; Boulon et al., 2008). Yeast R2TP components Rvb1/Rvb2 associate independently with whole RNA pol II and the Hsp90 complex (Lakshminarasimhan et al., 2016). R2TP subunits have also been detected in the polysomes interacting with newly synthesized Rpb1 subunits (Villanyi et al., 2014). Furthermore, the co-translational assembly between R2TP, Hsp90, and Rpb1 is mediated by Not5 (Villanyi et al., 2014). Similarly, in human cells, the co-chaperone R2TP complex works with HSP90 in the activation and assembly of several macromolecular complexes, including RNA pol II (Boulon et al., 2010). Proteomic analyses in human cells have evidenced the presence of RPB1-RPB8 dimer (RPB8 also called RPABC3) that interacts with the full R2TP/PFDL complex (Boulon et al., 2010). Human R2TP subunit RPAP3 delivers unassembled RPB1 to HSP90 and also associates with the largest subunits RPA190 (also called RPA1) (Boulon et al., 2010) and RPC160 (also called RPC1) of the free RNA pol I and RNA pol III (Jeronimo et al., 2007), respectively. These data suggest that R2TP subunit RPAP3 may be involved in the assembly of all three RNA pols (Boulon et al., 2010).

The human R2TP complex interacts with components of the prefoldin (PFD) complex (PFDN2 and PFDN6) and the prefoldin-like complex (URI, UXT, and PDRG1) to form the R2TP/prefoldin-like complex (R2TP/PFDL) (Cloutier et al., 2009; Cloutier and Coulombe, 2010; Martínez-Fernández et al., 2018). URI yeast ortholog Bud27 interacts with prefoldin subunits 2 and 6 (Pfd2 and Pfd6) and with the RNA pols common subunit Rpb5 and plays a role in the cytoplasmic assembly of RNA pol I, II, and III (Cloutier et al., 2009; Cloutier and Coulombe, 2010; Mirón-García et al., 2013; Martínez-Fernández et al., 2018).

These data suggest that R2TP, in both yeast and human, participates in the assembly of subassembly complex Rpb1 to the rest of the RNA pol II enzyme and likely occurs for RNA pol I and III.

### HSP90

Yeast Hsp90 and human HSP90 are well-conserved molecular chaperones that participate in protein folding and avoid the non-specific aggregation of non-native proteins (Pearl and Prodromou, 2006; Wandinger et al., 2008; Taipale et al., 2010; Makhnevych and Houry, 2012; Schopf et al., 2017).

As indicated above, yeast Hsp90 associates with RNA pol II (Lakshminarasimhan et al., 2016) and human HSP90 and R2TP/PFDL mediate the assembly of RNA pol II through the interaction with the RPB1 subcomplex (Boulon et al., 2010, 2012; Makhnevych and Houry, 2012). Interestingly, yeast Rpa135 (RNA pol I) and Rpc40 (RNA pol III) feature among Hsp90 clients (McClellan et al., 2007). These data suggest that Hsp90 could mediate the assembly of RNA pol I, II, and III. HSP90 is required for RPB1 stabilization through most of the assembly pathway, particularly for the RPB1 subunit association with RPB8 (also called RPABC3) and RPB5 (also called RPABC1) and also with the RPB2-RPB3-RPB10-RPB11-RPB12 subcomplex (also called RPB2-RPB3-RPABC5-RPB11-a and RPABC4), by facilitating the assembly of the complete enzyme (Boulon et al., 2010, 2012). Therefore, HSP90/R2TP could mediate a quality control mechanism for RNA pol II formation by ensuring its correct assembly before its nuclear import (Boulon et al., 2012). Interestingly, and as previously demonstrated for yeast (Lakshminarasimhan et al., 2016), proteomic analyses have also revealed not only RPB1 but also RPA190 (also called RPA1) and RPC160 (also called RPC1) to be HSP90/R2TP interactors, which also suggests their role in the assembly of RNA pol I and III (Boulon et al., 2010).

### Bud27

Bud27, and its human ortholog URI, are members of the PFD family of the ATP-independent molecular chaperones considered to function as scaffold proteins capable of assembling additional members of the PFD family in both human and yeast (Gstaiger et al., 2003; Martínez-Fernández and Navarro, 2018). Bud27 contacts the Pfd6 and Pfd2 components of the PFD/GimC complex (Gstaiger et al., 2003; Mirón-García et al., 2013), whereas URI contacts the PFD complex (Gstaiger et al., 2003).

Both Bud27 and URI interact with Rpb5, a common subunit of eukaryotic RNA pols (Dorjsuren et al., 1998; Gstaiger et al., 2003; Mirón-García et al., 2013; Martínez-Fernández and Navarro, 2018). Bud27 mediates the cytoplasmic assembly of the three RNA pols in *Saccharomyces cerevisiae* in an Rpb5-dependent manner before nuclear translocation, which probably occurs similarly for human URI, at least for RNA pol II (Mirón-García et al., 2013). Furthermore, proteomic analyses reveal URI to be an R2TP/PFDL component involved in, at least, RNA pol II assembly in human (Cloutier et al., 2009; Cloutier and Coulombe, 2010; Martínez-Fernández et al., 2018).

Bud27 shuttles between the nucleus and the cytoplasm (Mirón-García et al., 2013) and participates in the transcription mediated by the three RNA pols (Mirón-García et al., 2014; Vernekar and Bhargava, 2015; Martínez-Fernández et al., 2020). These results suggest that Bud27 could be imported to the nucleus in association with RNA pols and then remain associated with the transcriptional complexes, probably by a tripartite interaction with Rpb5 and remodeler complexes like RSC (Mirón-García et al., 2014; Vernekar and Bhargava, 2015).

### The GPN-Loop GTPase Family

GPN-loop GTPase proteins, highly conserved from archaea to humans, contain a highly conserved GPN-loop motif of Gly-Pro-Asn inserted into the GTPase core-fold that functions in GTP hydrolysis (Gras et al., 2007). In yeast, three GPN-loop GTPase, Npa3, Gpn2, and Gpn3 (GPN1, GPN2, and GPN3 in human, respectively), have been described, which evolved from a single archaeal counterpart (Gras et al., 2007; Forget et al., 2010; Carre and Shiekhattar, 2011; Staresincic et al., 2011; Minaker et al., 2013).

The three yeast and human small GTPases have been described to participate in RNA pol II assembly and/or transport to the nucleus (Forget et al., 2010; Carre and Shiekhattar, 2011; Staresincic et al., 2011; Minaker et al., 2013; Niesser et al., 2015; Li et al., 2018; Zeng et al., 2018; Liu et al., 2020). Furthermore, interactions among the three yeast and human members of the GPN-loop GTPase family have been detected (Uetz et al., 2000; Boulon et al., 2010; Forget et al., 2010; Carre and Shiekhattar, 2011; Staresincic et al., 2011; Minaker et al., 2013; Liu et al., 2020). A more detailed overview of these proteins is shown below.

#### Npa3

Npa3 interacts with RNA pol II and the R2TP complex in yeast (Forget et al., 2010; Niesser et al., 2015). Similarly, the human Npa3 ortholog, GPN1, has been described as an RNA pol II-associated protein that interacts with not only the complex R2TP/PFDL, and the cytosolic chaperonin, CCT, but also with other proteins involved in protein assembly and/or folding (Jeronimo et al., 2007; Forget et al., 2010). Npa3 depletion leads to the cytoplasmic accumulation of Rpb1 and Rpb3 (Staresincic et al., 2011). Cytoplasmic Rpb1 accumulation is also observed in mutants of the Npa3 GTP-binding domain or GPN motifs (similarly for human GPN1) (Forget et al., 2010; Carre and Shiekhattar, 2011; Staresincic et al., 2011). Nevertheless, Rpb3 immunoprecipitation evidences that Npa3 coordinates not only with Gpn3 but also with Rba50 (described in detail in the next paragraph), for the correct association of Rpb1 and Rpb2 with the Rpb3 subcomplex, likely in the cytoplasm (Liu et al., 2020). These data point to a major role of this protein in the cytoplasmic assembly of RNA pol II as has been previously proposed (Niesser et al., 2015). In line with this, two-hybrid assays have shown that the Rpb2 subunit contacts Npa3 and Rba50, which suggests that both proteins may coordinate the Rpb2 subcomplex-dependent assembly of RNA pol II (Liu et al., 2020). In fact, Npa3 is found mainly in the cytoplasm (Huh et al., 2003), but it contains a nuclear export sequence (NES) (Staresincic et al., 2011) that is also conserved in its human ortholog GPN1 (Reyes-Pardo et al., 2012). Npa3 and, similarly human GPN1, translocates to the cytoplasm by the action of the Xpo1/Crm1 pathway (Forget et al., 2010; Carre and Shiekhattar, 2011; Staresincic et al., 2011) and has been proposed to participate mainly in the nuclear import of RNA pol II (Forget et al., 2010; Carre and Shiekhattar, 2011; Staresincic et al., 2011). In line with data in yeast, two-hybrid experiments in human cells have demonstrated the interaction between GPN1 and Rba50 human ortholog RPAP1 and also between GPN1 and RPB2 (Liu et al., 2020). These findings suggest that the role proposed for Npa3 in the assembly of RNA pol II is also conserved in human cells.

#### Gpn2

Gpn2 is another GPN-loop GTPase family member (Alonso et al., 2013). Similar to Gpn1 and Gpn3, Gpn2 loss-of-function leads to altered cytoplasmic RNA pol II localization, which suggests a role for Gpn2 in the transport of RNA pol II to the nucleus (Staresincic et al., 2011; Minaker et al., 2013; Zeng et al., 2018; Liu et al., 2020). Gpn2 physically interacts with Rba50 and Rpb12 by cooperating to assemble the Rpb3 subcomplex prior to its association with Rpb1 and Rpb2 (Zeng et al., 2018). Yeast *gpn2* mutants show genetic interactions with RNA pol I and III mutants (Minaker et al., 2013). Although *gpn2* mutants affect the localization of both RNA pol II and III, they do not mislocalize RNA pol I subunits (Minaker et al., 2013). These results suggest that Gpn2 acts not only in RNA pol II assembly but also in the RNA pol I and III assembly process.

#### Gpn3

Gpn3 has been proposed to mediate RNA pols biogenesis, assembly, and transport to the nucleus (Minaker et al., 2013; Liu et al., 2020) and has been demonstrated to form a stable complex with Gpn1 in both yeast and human (Carre and Shiekhattar, 2011; Cristóbal-Mondragón et al., 2019; Liu et al., 2020). In addition, yeast *gpn3* mutants mislocalize RNA pol II and III subunits, which suggests a role for Gpn3 (and Gpn2) in not only RNA pol II but also in RNA pol III assembly and/or transport (Minaker et al., 2013). Gpn3 has been proposed to act upstream of import factor Iwr1 during RNA pol II biogenesis (Minaker et al., 2013). Proteomic analyses have evidenced the human GPN3, as well as GPN1 and GPN2, to be an interactor of the RNA pol II subunits (Forget et al., 2010; Calera et al., 2011). The human GPN1/GPN3 complex associates with RNA pol II in both the nucleus and the cytoplasm (Carre and Shiekhattar, 2011) and specifically binds RPB7 (likely the RPB7/RPB4 dimer) and the C-terminal domain (CTD) of RPB1 *in vitro* (Carre and Shiekhattar, 2011). Furthermore, the depletion of human *GPN3* or *GPN1* by small interfering RNAs (siRNAs) leads to RPB1 cytoplasmic accumulation (Calera et al., 2011; Carre and Shiekhattar, 2011). In line with a role for GPN3 in RNA pol II transport to the nucleus, the Q279^∗^ mutation of GPN3, related to cancer, has been described to lead to GPN3 entering the cell nucleus and inhibiting GPN1 nuclear export (Barbosa-Camacho et al., 2017).

By taking these data collectively, the GPN-loop GTPase family would act by favoring the assembly and/or transport of the three RNA pols in yeast, and these roles could be conserved in human cells.

### Rba50/RPAP1

Rba50 has been described as a cytoplasmic protein that interacts not only with the Rpb10 subunit in two-hybrid screening (Ito et al., 2001; Huh et al., 2003) but also with Rpb2, Rpb3, and Rpb11 in TAP-tagging analyses (Hazbun et al., 2003).

In yeast, physical and functional interactions have been demonstrated between Rba50 and the small GTPases Gpn2 and Npa3, and these interactions are conserved in human and *Arabidopsis* (Muñoz et al., 2017; Li et al., 2018; Zeng et al., 2018; Liu et al., 2020).

Recent studies propose the coordinated action between Rba50 and Gpn2 in the Rpb3 subcomplex assembly prior to its association with the Rpb2 subassembly complex (Zeng et al., 2018). The association among Rba50, Rpb3, Rpb10, and Rpb11 and between Gpn2 and Rpb12 would allow the Rpb3 subassembly complex formation (Zeng et al., 2018). Although yeast *rba50-3* mutant cells affect Rpb1 distribution, no interactions between Rpb1 and Rba50 or Gpn2 have been identified, which suggests that Rba50 and Gpn2 only transiently associate with the Rpb3 subcomplex and dissociate once Rpb1 is associated during the assembly of RNA pol II (Zeng et al., 2018). The recent observation that Rba50 and Npa3 not only interact but also target Rpb2 during the biogenesis of RNA pol II suggests that Rba50 also associates with Npa3 by increasing its affinity to Rpb2 to facilitate Rpb2 assembly to the previously formed Rpb3 subassembly complex (Liu et al., 2020). As previously indicated, human Rba50 ortholog RPAP1 interacts with GPN1, which associates with RPB2 (Liu et al., 2020). RPAP1 enters the nucleus and has been proposed to be required for the transcription of cell identity genes (those genes regulating the developmental process and fibroblastic/mesenchymal identity) by operating at the interface between the Mediator and RNA pol II (Lynch et al., 2018).

Based on yeast and human data, some authors propose that Rba50 (and probably its human ortholog RPAP1) functions by favoring a platform for other assembly factors like Gpn2 and Npa3 to sequentially mediate the association of RNA pol II subassembly complexes (Liu et al., 2020).

### Rtr1/RPAP2

Rtr1 (“regulator of transcription” 1) has been described as a phosphorylated RNA pol II interactor by acting as an S5-P CTD phosphatase during the transition from the initiation of the transcription to elongation *in vivo* (Gibney et al., 2008; Mosley et al., 2009, 2013; Hsu et al., 2014; Smith-Kinnaman et al., 2014; Hunter et al., 2016). Additional roles have been proposed for Rtr1 in transcription and mRNA stability (Mosley et al., 2013; Hsu et al., 2014; Hodko et al., 2016; Victorino et al., 2020) (our unpublished data).

It has been proposed that Rtr1 acts as a nuclear RNA pol II import factor as *RTR1* deletion causes cytoplasmic accumulation of Rpb1 and Rpb2 (Gómez-Navarro and Estruch, 2015). Rtr1 shuttles between the nucleus and cytoplasm in a Crm1-dependent manner (Gibney et al., 2008) and interacts with the nucleocytoplasmic transport protein Ran (a small Ras-like GTPase) (Braunwarth et al., 2003). The deletion of *RTR1* paralog *RTR2* does not cause mislocalization of Rpb1, but it increases in the double *rtr1 rtr2* mutant. These findings suggest that both proteins may play a redundant role in RNA pol II import (Gómez-Navarro and Estruch, 2015). Interestingly, Rtr1 also copurifies with the GTPases Gpn3 and Npa3 (Mosley et al., 2013; Smith-Kinnaman et al., 2014), which are proteins that have also been implicated in the nuclear import of RNA pol II (Staresincic et al., 2011).

Human Rtr1 ortholog RPAP2 has been proposed to act as an RNA pol II import factor, given its silencing results in RPB1 cytoplasmic accumulation (Forget et al., 2013). RPAP2 interacts with RNA pol II through its nuclear retention domain *in vitro* (Forget et al., 2013) and directly binds the RPB6 subunit of the enzyme (Wani et al., 2014). In addition, proteomic analyses of human subassembly complexes have identified that RPAP2 is preferentially associated with the free RPB3 and RPB1 subunits (Boulon et al., 2010), which suggests a role for RPAP2 in the biogenesis of RNA pol II. Furthermore, RPAP2 shuttles to the cytoplasm in association with GPN1 (Forget et al., 2013; Guerrero-Serrano et al., 2017). As in yeast, this evidences the relationship between RPAP2 and small GTPases. Interestingly, *Arabidopsis* Rtr1 ortholog RIMA interacts with MINIYO (the Rba50 yeast ortholog) (Muñoz et al., 2017).

By considering the role of Rtr1 in only the transcription of RNA pol II (Gibney et al., 2008; Mosley et al., 2009; Hsu et al., 2014; Victorino et al., 2020) and the above data, we speculate that this protein is specific to the assembly and/or transport of RNA pol II in yeast and also in other organisms.

### Rtp1

Rtp1 has been proposed to be an important factor for nuclear RNA pol II localization *via* Iwr1-independent pathways (Gómez-Navarro et al., 2013). In fact, it has been suggested to participate in transporting RNA pol II through the nuclear pore complex (Gómez-Navarro et al., 2013; Gómez-Navarro and Estruch, 2015). Rtp1 physically interacts with R2TP complex components and also with several RNA pol II subunits (Gómez-Navarro et al., 2013). Mass spectrometry data suggest that Rtp1 can facilitate the interaction between subassembly complexes Rpb2 and Rpb3, and their later interaction with the subassembly complex Rpb1 for the assembly of RNA pol II (Gómez-Navarro et al., 2013). *RTP1* gene depletion in yeast leads to the cytoplasmic accumulation of Rpb1 and Rpb2 (Gómez-Navarro et al., 2013). Interestingly, the fact that the *rtp1* mutant shows no clear cytoplasmic accumulation of small RNA pol II subunits, such as Rpb3 and Rpb11, suggests the existence of passive nuclear diffusion of small RNA pol II subunits (Gómez-Navarro and Estruch, 2015).

### Iwr1

Iwr1 was initially reported as a protein that interacts with almost every RNA pol II subunit to regulate the transcription of some genes (Gavin et al., 2002; Peiro-Chova and Estruch, 2009) and was later reported as being important for preinitiation complex formation by all three nuclear RNA pols in *S. cerevisiae* (Esberg et al., 2011). It has been proposed that the main pathway for the nuclear RNA pol II import involves yeast Iwr1 (Czeko et al., 2011). Iwr1 binds the cleft of the active center of RNA pol II once the enzyme is fully assembled and uses its NLS signal to direct the nuclear import of RNA pol II (Czeko et al., 2011). Iwr1 is displaced from active RNA pol II in the nucleus, which facilitates its export and recycling (Czeko et al., 2011; Wild and Cramer, 2012). Nevertheless, *iwr1*Δ mutant strains show cytoplasmic accumulation of both Rpb1 and Rpb3 (Czeko et al., 2011), which suggests interferences with the assembly of RNA pol II.

In addition to Iwr1-dependent nuclear RNA pol II import, Iwr1-independent import pathways have been proposed as mechanisms that maintain cell viability when the main pathways are blocked, which involves the nuclear import of individual or partially assembled subunits, even by diffusion (Gómez-Navarro and Estruch, 2015).

### Rbs1

Rbs1 was originally identified as a PAS kinase suppressor in a genetic high-copy suppressor study (Rutter et al., 2002). More recently, a role for Rbs1 in RNA pol III assembly and transport to the nucleus has been proposed (Cieśla et al., 2015). This role for Rbs1 has also been described in another article published in the same issue by Boguta and Turowski (in press). Rbs1 physically interacts not only with several RNA pol III subunits, such as Rpc19 and Rpc40 but also with the RNA pol common subunit Rpb5 (Cieśla et al., 2015). Rbs1 has been described as a Crm1 interactor, and it shuttles between the nucleus and the cytoplasm. Accordingly, it has been suggested to likely interact with the RNA pol III complex to mediate its nuclear translocation (Cieśla et al., 2015). Rbs1 has also been proposed to mediate the biogenesis of RNA pol III by controlling the steady-state levels of *RPB10* mRNA by interacting with its 3′ UTR region (Cieśla et al., 2020).

## Concluding Remarks

Despite the pre-existing knowledge about RNA pols assembly in yeast and their transport to the nucleus, mainly focused on RNA pol II and likely well-conserved for human RNA pol II, many interesting questions still need answers: although the assembly of RNA pols in eukaryotes seems to be similar to that in bacteria, why do RNA pols-specific processes exist? Why do RNA pol-specific assembly factors exist, while others seem to be general? Can different and additional mechanisms act for the assembly of RNA pols in yeast and other eukaryotes? Which mechanisms account for the nuclear transport of different RNA pols?

Resolving these important questions and others must be the goal of future studies, to help us better understand the mechanisms governing the assembly and nuclear transport of different RNA pols.

## Author Contributions

All authors wrote and reviewed this manuscript.

## Conflict of Interest

The authors declare that the research was conducted in the absence of any commercial or financial relationships that could be construed as a potential conflict of interest.
